# Cardiac arrest secondary to subclavian artery injury in blunt chest trauma: A lifesaving emergency surgery in COVID crises

**DOI:** 10.1016/j.amsu.2022.103454

**Published:** 2022-03-07

**Authors:** Ikram ul Haq Chaudhry, Othman M Al Fraih, Meenal A Al Abdulhai, Hisham Al Maimon, Yousif A Alqahtani, Mohammad Tariq khan, Abdullah M Al Ghamdi

**Affiliations:** Division of Thoracic Surgery Dammam Medical Complex, Dammam, 31444, Saudi Arabia

**Keywords:** Blunt trauma, Subclavian artery, Cardiac arrest, Surgery

## Abstract

A 25-year-old male vehicle driver had a road traffic accident and sustained a blunt chest injury. His chest x-ray in the emergency department showed left hemithorax opacification. A chest drain Fr32 was inserted, and 1300ml of Blood drained out. While having a computed tomographic scan of the thorax scan, he had a cardiac arrest and after Cardiopulmonary Resuscitation (CPR) he was transferred to our tertiary care hospital on a mechanical ventilator and massive ionotropic support (adrenaline and noradrenaline) with a blood pressure of 50/24 mmHg. We performed a lifesaving emergency thoracotomy in a supine position with all COVID precautions, as COVID status was not available before hospitalization. After the repair of the Subclavian artery patient recovered completely and was discharged for follow-up in outpatient.

## Highlights

• A 25-year-old male driver sustained blunt chest injury.
• Chest Ray showed a left hemithorax opacification. Chest drain was inserted that drained 1000 ml of blood.
• Patient had cardiac arrest while doing CT scan. He was intubated and put on mechanical ventilation. Inotropes infusion was started patient was transferred to our tertiary care hospital in very unstable condition.
• Lifesaving surgery in such emergency condition with full Covid protection gear over the scrub suits is very challenging for surgeon in addition to unfavorable clinical condition of a patient.

## 1 Introduction

Blunt traumatic Subclavian artery injuries (TSAI) are rare. These vessels are well protected by their complex anatomy and musculoskeletal tunnel formed by subclavius muscle, clavicle, first rib, deep cervical fascia, and cost coracoid ligament. Subclavian artery injuries represent 2% of all acute vascular injuries, it accounts for 1–5% of all vascular trauma [1,2]. These injuries are less common in children due to more elasticity of the chest wall and vessels. Most of the patients die before reaching the hospital. Subclavian artery injuries are caused by two mechanisms elongation and stretching or laceration. In blunt chest trauma, applying force to the clavicle or anterior shoulder, as it happens in motor traffic accidents, leads to an extension of a subclavian vessel. Bony fragments from fracture of the clavicle, first rib, and scapula can cause laceration of subclavian vessels [3]. Fracture of the clavicle is associated with 50% of cases of subclavian artery injuries. This case is reported in line with scare criteria [4].

## 2 Case

**Fig. 1:**
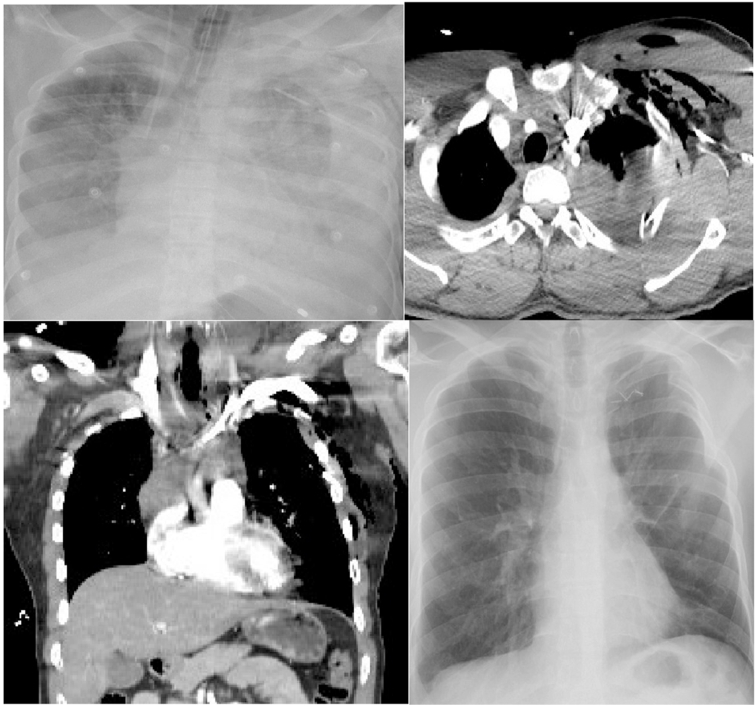
(A) Chest x-ray showing left hemothorax and chest drain. (B) CT scan of thorax axial view showing leakage of contrast in left upper chest most likely from subclavian artery injury and hemothorax (c) CT scan thorax coronal view showing some contrast leak (D) Post operative Chest -ray unremarkable showing some surgical clips.

A 25-year-old male vehicle driver had a road traffic accident and sustained blunt chest injuries. Chest X-ray showed opacification of left hemithorax, a chest drain was inserted, and 1300 ml of Blood drained Fig. 1(A). The patient had a cardiac arrest in the referral hospital while doing a CT scan of the chest Fig. 1(B&C). He received cardiopulmonary resuscitation and was transferred to our tertiary care hospital on mechanical ventilation and massive inotropes (adrenaline and noradrenaline). On arrival, his blood pressure was 50/24 mmHg on 35 μg of noradrenalin and 25 μg of adrenaline. Arterial Blood gases showed metabolic acidosis at pH 6.2, PCO_2_ 36.2, PO_2_ was 90 on hundred percent Fio_2%_, and PEEP of 15. A basic blood investigation revealed, Hemoglobin 7.1/dl and normal renal, hepatic panels. He received 3 L of normal saline before surgery.

Ten units of Blood, six units of platelets, and fresh frozen plasma were arranged. Emergency lifesaving Left anterior thoracotomy was carried out through 3rd intercostal space in the supine position on two lung ventilation as the single lung ventilation was not possible due to hemodynamic instability. The left chest was full of clots and Blood. After clearing all the clots, the left hemithorax apex area was packed with surgical gauze to apply compression in the left subclavian artery. Blood pressure increased to 100 after transfusion of six units of Blood. On removing the surgical pack, we found bleeding from the partial tear at the origin of the left subclavian artery was repaired with a 4/0 proline.

Further, four units of Blood, platelets, and FFP were transfused. One 32 Fr chest drain was inserted, and the chest closed in layers. The patient was transferred to ICU. Inotropes were weaned off gradually, and the patient was extubated two days later and moved to the ward. A chest drain was removed on the 4th postoperative day, and the patient was discharged on day nine for follow-up in outpatient. His postoperative chest X-ray was unremarkable Fig. 1(D). His left radial pulse was regular, and the pulse oximeter showed 99% oxygen saturation.

## 3 Discussion

Although subclavian artery injury after a blunt is rare due to its anatomical location and musculoskeletal protection in an anatomical grove, it is a lethal condition with a terrible prognosis. Most of the patients die before reaching the hospital. Rulliat et al. reported TSAI incidence among 1181 thoracic trauma patients was 0.4% [5]. Rich et al. reported that the incidence of subclavian arterial injury was 1% after the analysis of the Vietnam War vascular registry [6]. Costa et al. studied 167 patients with mediastinal arterial injuries and reported 15 patients had subclavian artery injury due to fracture of the first rib [7]. In another study reported by Philips, out of 45 patients with first rib fracture due to blunt trauma, they found four of the patients had subclavian artery injury [8]. Precise diagnosis of the subclavian artery is challenging.

Therefore, subclavian vessel injury should be ruled out carefully in polytrauma patients. Patients sustaining subclavian artery injury may have minimal symptoms initially as less than 25% of patients had any significant sign-on presentation [9]. Sturm and Cicero et al. reported a clinical complex of five things for suspecting subclavian vessels injuries, including fracture of the first rib, palpable supraclavicular hematoma, and widened mediastinum or chest x-ray demonstrating apical radio density a sign of hematoma over subclavian vessel site, absent radial pulse, and brachial plexuses palsy [10]. In medical literature, 32–59% of patients with traumatic subclavian vascular injuries had pulse deficits [11]. Careful clinical examination of the upper limb including, color, temperature, sensation, hand motility, a radial pulse, is mandatory. Only 20% of patients had hard signs, and pulse deficit is found in only 32–59% of patients [12]. CT scan with contrast is the best diagnostic modality; in selective stable patient's arteriography is a handy tool to precisely locate the site of injury and plan the surgical approach [13].

**Fig. 2:**
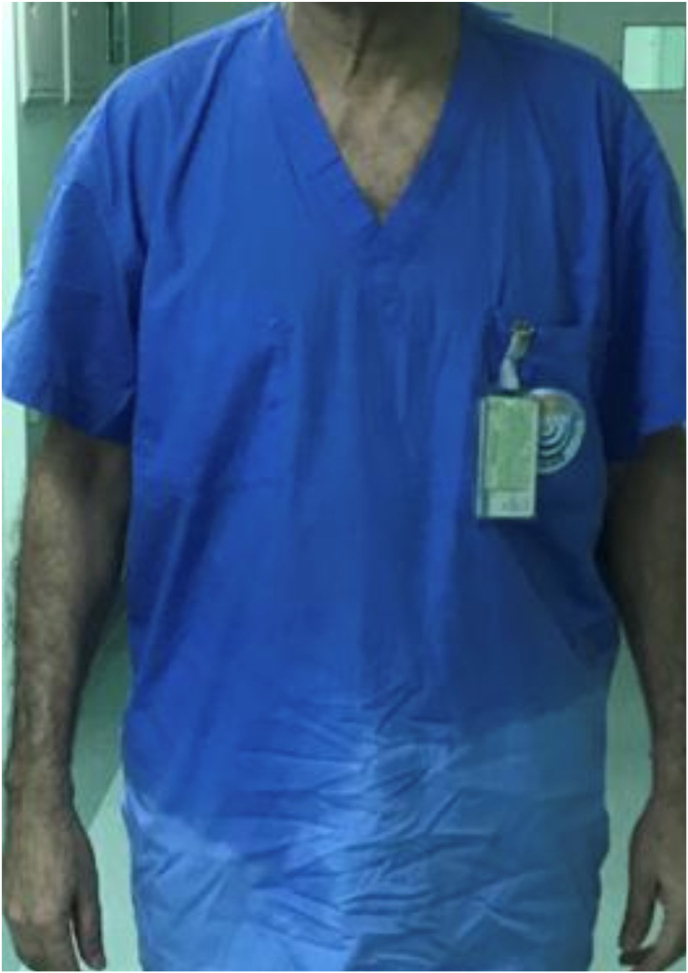
Surgeon picture after the completion of surgery. We performed surgery having COVID protective gear over scrub suit.

DeMeules, Cramer, and Perry (1971) reported criteria for angiography including. If there is evidence of widened mediastinum, a fractured sternum, multiple rib fractures with crushed chest, first rib fracture, posteriorly displaced clavicular fracture, peripheral pulse deficit, unexplained hypotension following adequate blood replacement, selected patients with massive Hemothorax or continued bleeding from chest tubes [14,15]. Management of traumatic subclavian artery injury depends on the severity (hemodynamic stability) and injury site. The conventional surgical approach is preferred in lifesaving situations. At the same time, the Endovascular procedure is less invasive, and the success rate is high if an injury is focal and traversed safely with a guidewire [16,17]. Last 24 years of review of the medical literature, 750 cases of SAI have been reported. Only 10.5% underwent endovascular repair. Another study of 569 patients reported that only eight cases (1.4%) underwent endovascular treatment. With the growing experience of vascular surgeons and recent advances in technology, the endovascular approach has been increasingly used in clinical practice for, past twelve years. Analysis showed that (39.2%) of patients with traumatic Subclavian injuries were treated with an endovascular approach. The endovascular technique has several advantages over conventional surgery, less postoperative recovery time,.limited dissection, less pain, less incidence of bleeding and wound infection. Endovascular approach results and post-intervention graft patency are limited to small series and case reports. This method has many complications, including access site hematoma formation, pseudoaneurysm, stent-graft stenosis, endoleak, thrombosis occlusion of the vertebral artery. Some patients eventually need open surgery [18,19]. The conventional surgical approach is used in lifesaving situations and when the endovascular procedure cannot be executed. Choice of the incision depends on the side and site of arterial injury. A standard left thoracotomy is used for left subclavian artery injuries, median sternotomy, and supraclavicular, and trap door incision has been used according to the site of injury and control of bleeding [20,21]. The mortality rate of subclavian artery injury ranges from 3 to 33%. Operative mortality of 14.3% has been reported in a series of 15 patients with subclavian artery injury, which is in parallel with other series [[22], [23], [24]]. Emergency lifesaving thoracotomy is an unstable patient in COVID pandemic where COVID status of the patient is not known is hugely challenging for an operating surgeon as the COVID protective gear over scrub suit is very uncomfortable, can cause profuse sweating and dehydration. Fig. 2.

## 4 Conclusion

We report a rare case of TSAI with left Hemothorax and cardiac arrest while having a CT scan of the thorax on table cardiopulmonary resuscitation was done. The patient came to hospital on mechanical ventilation and massive inotropic support. In a supine position, lifesaving anterior thoracotomy was performed above the nipple line, and exposure was difficult because the patient was not stable for single-lung ventilation due to hemodynamic instability. The primary repair of the subclavian artery tear was carried out successfully. The patient recovered fully and was discharged home. Emergency surgery in a supine position and without single ventilation with a complete COVID protection kit is very challenging for the surgeon. The patient had unremarkable recovery and resumed back to his normal life.

## Patient consent

Written informed consent was obtained from the patient for publication of this case report and accompanying images. A copy of the written consent is available for review by the Editor-in-Chief of this journal on request”.

## Sources of funding

No source of funding in this case.

## Ethical approval

IRB Approval.

## Provenance and peer review

Not commissioned, externally peer reviewed.

## Declaration of competing interest

No conflict of interest.
